# Obesity promotes the expansion of metastasis-initiating cells in breast cancer

**DOI:** 10.1186/s13058-018-1029-4

**Published:** 2018-09-04

**Authors:** Mélanie Bousquenaud, Flavia Fico, Giovanni Solinas, Curzio Rüegg, Albert Santamaria-Martínez

**Affiliations:** 1 Experimental and Translational Oncology Laboratory, Division of Pathology, Department of Oncology, Microbiology and Immunology, Faculty of Science and Medicine, University of Fribourg, Fribourg, Switzerland; 2 Tumor Ecology Laboratory, Division of Pathology, Department of Oncology, Microbiology and Immunology, Faculty of Science and Medicine, University of Fribourg, Chemin du Musée 18, PER17, CH-1700 Fribourg, Switzerland; 3 Department of Molecular and Clinical Medicine, The Wallenberg Laboratory, University of Gothenburg, Gothenburg, Sweden; 4 Swiss Integrative Center for Human Health, Fribourg, Switzerland

**Keywords:** Obesity, Breast cancer, Metastasis-initiating cells

## Abstract

**Background:**

Obesity is a strong predictor of poor prognosis in breast cancer, especially in postmenopausal women. In particular, tumors in obese patients tend to seed more distant metastases, although the biology behind this observation remains poorly understood.

**Methods:**

To elucidate the effects of the obese microenvironment on metastatic spread, we ovariectomized C57BL/6 J female mice and fed them either a regular diet (RD) or a high-fat diet (HFD) to generate a postmenopausal diet-induced obesity model. We then studied tumor progression to metastasis of Py230 and EO771 grafts. We analyzed and phenotyped the RD and HFD tumors and the surrounding adipose tissue by flow cytometry, qPCR, immunohistochemistry (IHC) and western blot. The influence of the microenvironment on tumor cells was assessed by performing cross-transplantation of RD and HFD tumor cells into other RD and HFD mice. The results were analyzed using the unpaired Student *t* test when comparing two variables, otherwise we used one-way or two-way analysis of variance. The relationship between two variables was calculated using correlation coefficients.

**Results:**

Our results show that tumors in obese mice grow faster, are also less vascularized, more hypoxic, of higher grade and enriched in CD11b^+^Ly6G^+^ neutrophils. Collectively, this favors induction of the epithelial-to-mesenchymal transition and progression to claudin-low breast cancer, a subtype of triple-negative breast cancer that is enriched in cancer stem cells. Interestingly, transplanting HFD-derived tumor cells in RD mice transfers enhanced tumor growth and lung metastasis formation.

**Conclusions:**

These data indicate that a pro-metastatic effect of obesity is acquired by the tumor cells in the primary tumor independently of the microenvironment of the secondary site.

**Graphical abstract:**

Effects of postmenopausal obesity on primary breast cancer tumoursᅟ
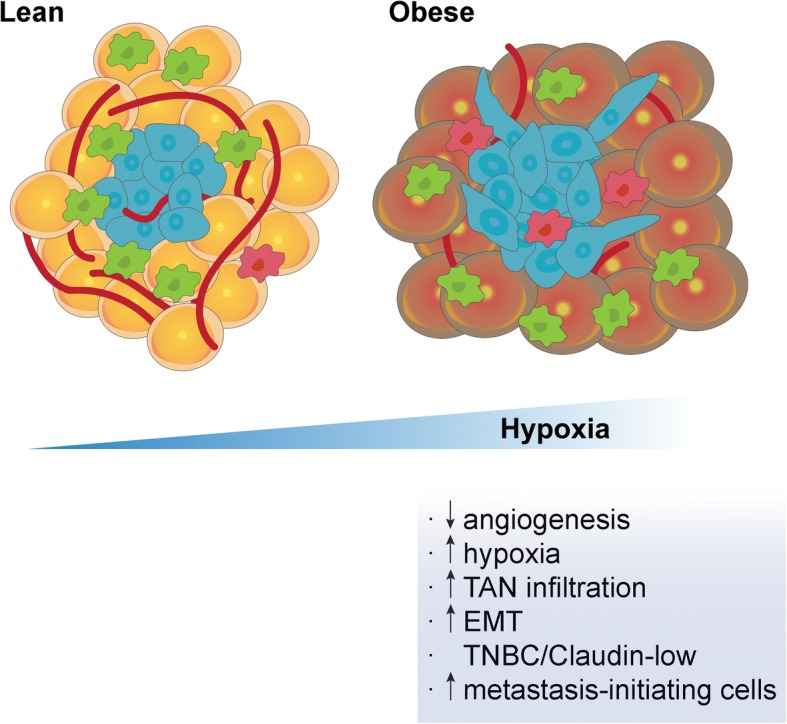

**Electronic supplementary material:**

The online version of this article (10.1186/s13058-018-1029-4) contains supplementary material, which is available to authorized users.

## Background

Obesity affects more than half a billion adults worldwide and is a well-known risk factor for many cancers, including breast cancer [1], showing correlation with both increased risk and poor prognosis [2]. Of note, this association is mainly linked to postmenopausal patients, whereas in premenopausal women increased BMI correlates with decreased breast cancer risk - yet more aggressive progression and resistance to therapy [3]. However, the biology behind these links remains unclear, in part due to the wide range of conditions associated with obesity.

Obesity-derived systemic complications, including but not restricted to inflammation, insulin resistance and hyperglycemia have been explored as potential causative effects or contributors to increased breast cancer risk and progression, albeit with mixed results [4]. Obesity is commonly characterized by macrophage-induced chronic inflammation in the adipose tissue [5, 6]. The effector cells leading to adipose tissue inflammation are M1 macrophages [7], which are initially recruited by T cells as monocytes [8]. Macrophages proliferate locally in the adipose tissue, a process that results in local and systemic subclinical inflammation leading to insulin resistance, diabetes and further increased adiposity [9]. Recent studies suggest that macrophages promote tumor progression in obesity through interactions with adipocytes [10], although M1 macrophages typically play protective roles in tumor formation [11]. Still, none of these studies provide experimental evidence to explain why obesity correlates with increased risk of distant metastasis, particularly in postmenopausal women [12]. Recently, two groups have found obesity to promote metastasis by two independent tumor cell extrinsic mechanisms [13, 14]. However, we and others have previously shown that metastasis relies on both tumor cell extrinsic and intrinsic factors [15]. With the aim to understand the molecular mechanisms linking obesity and poor prognosis in postmenopausal breast cancer, we generated a syngeneic orthotopic mouse model of postmenopausal breast cancer and investigated the effects of obesity on primary tumor growth and spontaneous metastatic progression. Our results reveal a novel mechanism involving hypoxia and neutrophil granulocytes-tumor cell interactions in the primary tumor that leads to the expansion of metastasis-initiating cells collectively resulting in increased distant metastasis formation.

## Methods

### Mouse work

C57BL/6 J, FVB/N, MMTV-PyMT (FVB/N) [16], and B6(Cg)-*Rag2*^*tm1.1Cgn*^/J (Rag2−/−) [17] mice were housed in ventilated cages in the mouse husbandry of the University of Fribourg. For tumor cell grafting, cells were trypsinized, resuspended in complete medium and centrifuged at 1300 rpm. They were washed twice in PBS, counted and resuspended in 1:3 Matrigel:PBS for injection into the 4^th^ mammary fat pad. To mimic postmenopausal estrogen decrease, 5–7-week-old female mice were ovariectomized and 2 weeks later they were supplied with either a high-fat diet (HFD) or a normal (regular) diet (RD (60% and 10% fat content, respectively). Mice were treated with clodronate liposomes as previously descrived [18]. All the experiments were performed by trained researchers holding the necessary accreditations and in accordance to the Swiss Animal Welfare Regulations and approved by the Cantonal Veterinary Service of the Canton Fribourg (2015_07_FR).

### Antibodies and reagents

The following antibodies and reagents were used: TER119, CD3 (17A2), CD4 (GK1.5), CD8a (53–6.7), CD19 (6D5), CD31 (MEC13.3), CD45 (30-F11), Ly6C (HK1.4), Ly6G (RB6-8C5), CD11b (M1/70) (Biolegend), CD31, PCNA (Santa Cruz Technologies), Cytokeratin 14 (Covance), CD11b, CD31, Ki67 (Abcam), α-SMA, β-Tubulin, β-Actin (Sigma), Vimentin (Lifespan Biosciences), N-Cadherin, E-cadherin, p21, p53 (Cell Signaling), hypoxia inducible factor 1 alpha (HIF1α) (Novus Biologicals) and PIMO (Hypoxiprobes).

### Cell culture

EO771 [19] and Py230 [20] cell lines were obtained from the American Type Culture Collection (ATCC) and grown as recommended. Mouse tumor tissue was dissociated using a mixture of Liberase TH (Roche) and DNAse at 37 °C for 45 min. Cells were filtered, washed twice in 2 mM EDTA in PBS and twice in PBS and then seeded for culture.

### Fluorescence-activated cell sorting (FACS) analysis

For FACS analysis, tumor cells derived from tumor grafts (Py230 and EO771) or primary MMTV-PyMT tumors were obtained by disaggregating the tumors with Liberase TH (Roche) and DNAse at 37 °C for 45 min with agitation. Cells were then washed, filtered, stained with the appropriate antibodies for 30 min at 4 °C; 4',6-diamidino-2-phenylindole (DAPI) was used to stain and discard dead cells. Fluorescence was analyzed using a MACSQuant (Miltenyi) analyzer. FACS data were processed and analyzed using FlowJo.

### Immunohistofluorescence

Immunostaining was performed on 4-μm-thick paraffin sections. Antigen retrieval was induced by heating the samples to 95 °C for 30 min in citrate buffer, pH 6.0. After blocking, we incubated the sections with the indicated antibodies overnight at 4 °C, and then used the secondary fluorescently labeled antibodies Alexa Fluor 488, 567 and 647 (Molecular Probes, Invitrogen) or HRP-conjugated secondary antibodies (Dako). Fluorescent images were taken with a TCS-SP5 confocal microscope (Leica). Light images were taken with a widefield microscope (Leica).

### Western blot

Protein was extracted with complete radioimmunoprecipitation assay (RIPA) buffer, separated by electrophoresis, transferred to polyvinylidene fluoride (PVDF) membranes, blocked with 5% BSA and incubated overnight with primary antibodies. Immunoreactive bands were visualized using HRP-conjugated secondary antibodies (Cell Signaling).

### Real-time PCR

RNA was prepared using the mini RNeasy kit (Qiagen). Complementary DNAs (cDNAs) were generated using oligo-T priming and the M-MLV transcriptase (H-) point mutant (Promega) and quantitative PCR (qPCR) was performed in a StepOnePlus thermocycler (Applied Biosystems) using the SYBR green PCR Master Mix (Kapa). A list of the primers used is shown in Additional file 1: TableS1.

### Statistics

Data were analyzed using GraphPad Prism 6. Means were compared using the unpaired Student *t* test. Samples were analyzed using Mann-Whitney’s non-parametric test if the data were not normally distributed (with normality assessed using the D’Agostino-Pearson omnibus normality test). When comparing more than two variables, we performed one-way or two-way analysis of variance (ANOVA). To isolate differences between groups in ANOVA, we performed Fisher’s least significant difference (LSD) test. We tested correlation using Pearson’s correlation coefficient or Spearman’s nonparametric correlation analysis depending on the data distribution. The *p* values are indicated for each experiment. Error bars in the figures indicate standard deviation unless stated otherwise in the figure legends. Significant differences between experimental groups are indicated with asterisks as follows: *p < 0.05, **p < 0.01, ***p < 0.001 and ****p < 0.0001.

## Results

### Mice fed with a HFD experience faster tumor growth and progression to metastasis

**Fig. 1:**
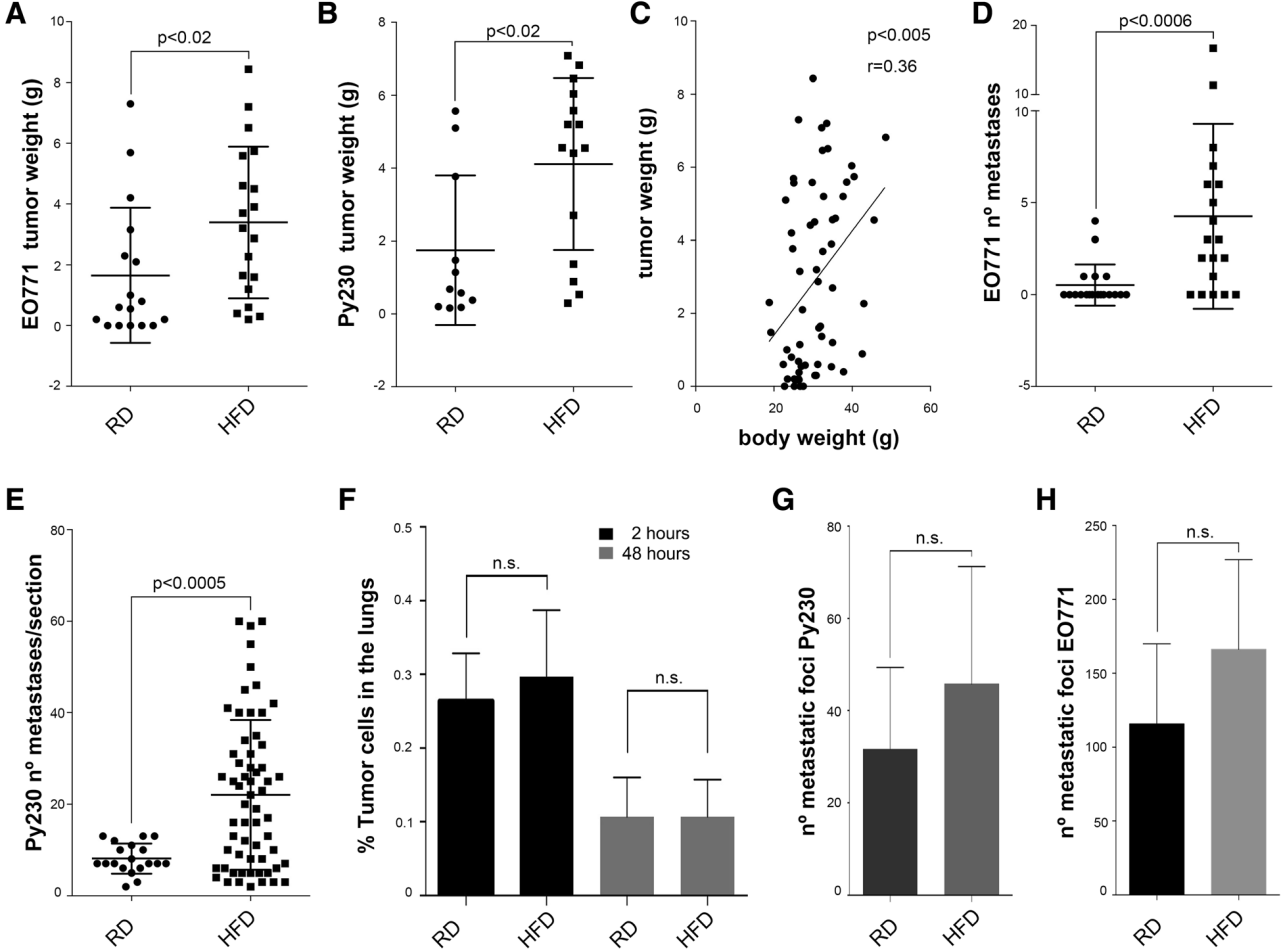
Effects of a high-fat diet (HFD) on tumor progression in mice. The tumor weight is increased in the HFD groups in both EO771 grafts (**a**, *n* = 14 regular diet (RD) and *n* = 16 HFD) and Py230 grafts (**b**, *n* = 11 RD and *n* = 15 HFD). Tumor weight correlates with body weight (**c**, *N* = 60). The number of lung metastases is increased in both EO771 tumor-bearing mice (**d**, *n* = 14 RD and n = 16 HFD) and Py230 bearing mice (**e**; *n* = 19 sections, RD and *n* = 61 sections, HFD). Py230 cells have the same extravasation capabilities in both obese and lean mice as seen by the percentage of Celltracker-labeled cells in the lungs at 2 h (*n* = 3 RD and *n* = 3 HFD) and 48 h (*n* = 4 RD and *n* = 4 HFD) by FACS (**f**). The number of metastatic colonies is also not changed in the RD compared to the HFD groups in Py230 (**g**, *n* = 4) or E0771 (**h**, *N* = 11)

In order to recapitulate postmenopausal obesity and assess how it affects breast cancer progression, we first generated an experimental model following the strategy schematically depicted in Additional file 2: Figure S1A. Ovariectomizing C57BL/6 J mice and feeding them with a high-fat diet (HFD, 60% fat content) significantly increased weight gain compared to non-ovariectomized HFD-fed mice and ovariectomized or non-ovariectomized mice fed with a regular diet (RD) (Additional file 2: Figure S1B). Between 20 and 25 weeks of age, the difference between the mean of the final weight in both groups was 39.7% (Additional file 2: Figure S1C). In addition, obese mice developed the common systemic conditions frequently observed in the HFD mouse model, such as hyperinsulinemia (data not shown) [21]. Obesity is mainly associated with estrogen receptor alpha-positive (ERα^+^) breast tumors [22]. To mimic human disease, we next performed syngeneic transplants in the mammary fat pad of C57BL/6 J mice with two different murine breast cancer cell lines that are hormone-sensitive in vivo, EO771 and Py230 [23, 24], and studied primary tumor growth and progression. As shown in Fig. 1a and b, E0771 and Py230 tumors in the HFD group grew significantly bigger. As with humans, in rodents the susceptibility to gain weight in response to obesogenic diets differs substantially between individuals [25, 26]. This variability is reflected within our experimental groups, since neither the RD nor the HFD body weights follow a normal distribution but are negatively and positively skewed, respectively (*p* < 0.0068; *n* = 29 and *p* < 0.007; *n* = 35, Additional file 2: Figure S1D and E). Nevertheless, our analyses revealed that body weight moderately correlated with tumor mass (Fig. 1c), which is again in agreement with observations in humans [27]. Interestingly, metastasis was also significantly increased in obese mice (Fig. 1d, e), even when there was no significant correlation between the size of the tumor and the number of metastatic foci in our control groups (*r* = 0.29, *p* = 0,22). Comparing same-sized tumors rendered similar results (Additional file 2: Figure S1F and G). To understand whether this increase in metastasis was due to tumor or host derived factors, we injected Celltracker-labeled Py230 cells into the tail vein of lean and obese mice and studied lung colonization using FACS after 2 h, as the time point for initial tumor cell trapping/seeding, and after 48 h, when most cells have extravasated. Our results show that there are no major differences in initial seeding and extravasation in lean compared to obese mice (Fig. 1f). Furthermore, we did not observe significant differences in the number of metastatic colonies formed upon tail vein injection, though there was a slight, non-significant trend toward more metastasis formation in obese mice (Fig. 1g, h). Taken together, these results demonstrated that obesity in ovariectomized mice promotes the formation of larger tumors and increased lung metastasis formation in the two models tested.

### Obesity and not dietary factors is responsible for differences in tumor progression

Recent clinical data suggest that a diet rich in unsaturated fatty acids correlates with breast cancer risk independently of body mass index (BMI) [28], particularly in postmenopausal women [29]. However, it is still not clear whether the diet itself contributes to a poor prognosis in those patients with breast cancer or whether obesity is required. We then aimed at assessing whether the effects observed on tumor growth and metastasis in our model were due to obesity or to the diet. It is well-known that alternatively activated macrophages (M2) protect against obesity and insulin resistance [30]. We therefore reasoned that using M1/Th1 and M2/Th2-biased mouse strains [31] would allow us to discriminate the relevance of the diet versus obesity in our setting. Hence, we switched to the FVB/N mouse strain, an archetypical M2/Th2-biased mouse strain, in which we could use the PyMT tumor model for consistency purposes. We ovariectomized female mice, fed them with either a RD or HFD and performed syngeneic transplants with MMTV-PyMT tumor-derived cells.

**Fig. 2:**
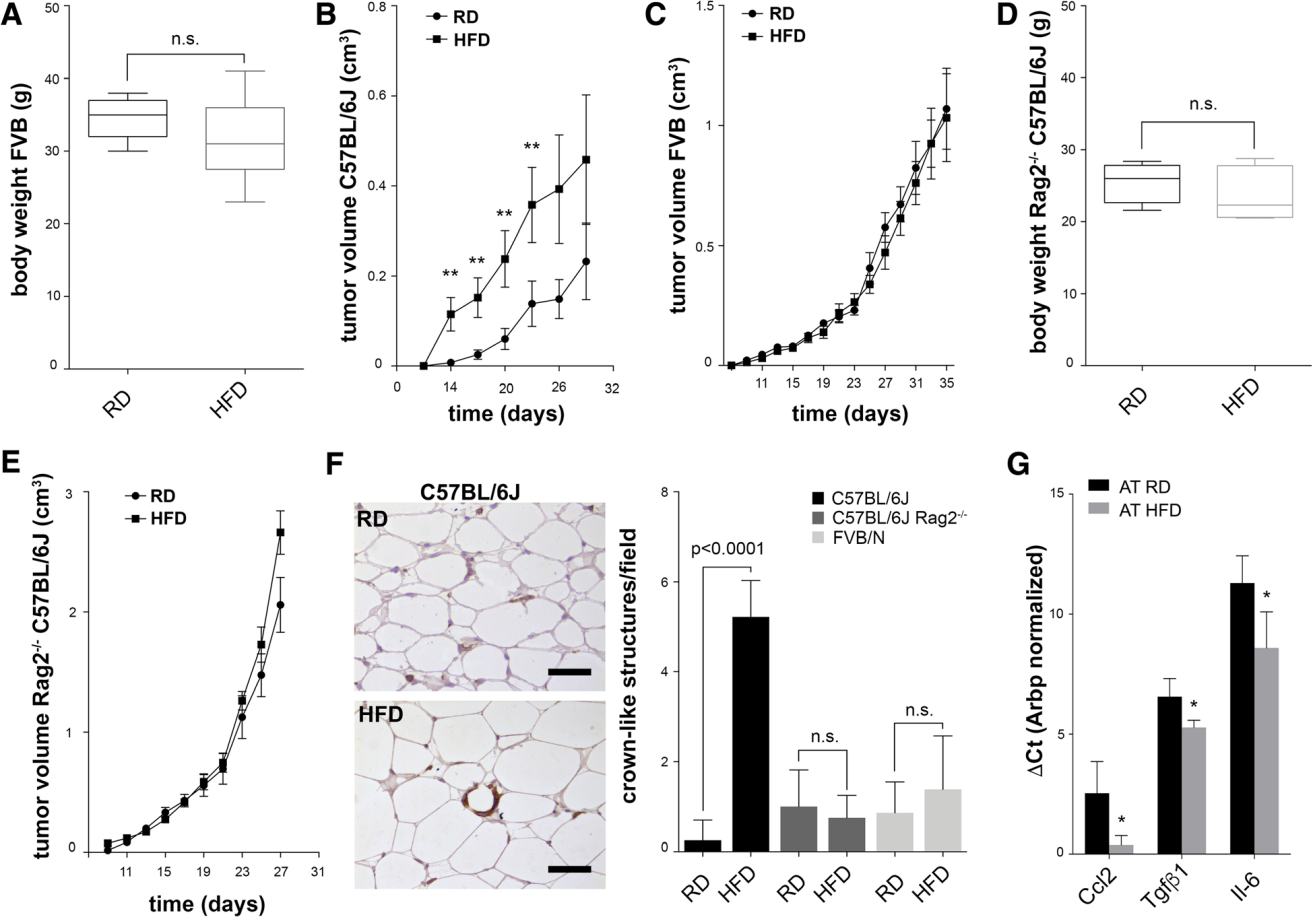
Obesity and not a high-fat diet (HFD) is responsible for tumor progression. FVB/N mice do not gain weight after 13 weeks of HFD (**a**, *n* = 4 regular diet (RD) and *n* = 5 HFD). Py230 tumors in C57BL/6 mice grow significantly faster in obese mice (**b**, *n* = 40), while in FVB/N mice PyMT tumors do not differ in growth dynamics between the RD and HFD groups (**c**, *n* = 4). C57BL/6 Rag2−/− mice do not gain weight on a HFD (**d**, *n* = 4 RD and *n* = 5 HFD), nor do Py230 tumors differ significantly in their growth dynamics when grafted in C57BL/6 Rag2−/− mice (**e**, *n* = 4 RD and *n* = 5 HFD). Immunohistochemical analysis of CD11b in the adipose tissue of RD and HFD C57BL/6 mice (scalebar 50 um) and quantification of crown-like structures in C57BL/6, FVB/N and C57BL/6 Rag2−/− mice (**f**). Quantitative PCR analysis of indicated targets in the adipose tissue of RD and HFD mice (**g**). Error bars in panels **b**, **c** and **e** indicate SEM. Ct, cycle threshold; Arbp, acidic ribosomal phosphoprotein P0

Our results show that FVB/N mice did not gain weight after 12 weeks on the HFD regimen (Fig. 2a). Contrary to Py230-injected C57BL/6 mice, in which RD and HFD tumor growth rates diverge very early (Fig. 2b), we found that FVB/N mice, tumors did not differ in growth kinetics between the RD and HFD groups (Fig. 2c). Recruitment and activation of resting macrophages into proinflammatory ones in the adipose tissue requires previous infiltration by CD8^+^ effector T cells [8]. Therefore, we argued that the absence of lymphocytes in an M1/Th1 biased strain should be sufficient to prevent obesity and rescue the obesity-mediated effects on tumor growth depicted in Fig. 1. Indeed, our results demonstrate that C57BL/6 J Rag2^−/−^ mice, which lack T and B cells but not macrophages, did not become obese after 12 weeks of HFD (Fig. 2d). Consistent with the lack of T cells, overall tumor growth was faster in C57BL/6 Rag2^−/−^ mice than in FVB/N mice. However, Py230 tumors did not progress faster in C57BL/6 Rag2^−/−^ mice fed with HFD compared to RD controls (Fig. 2e). Moreover, in contrast to HFD-fed wild-type C57BL/6 J controls, the peritumoral adipose tissue of HFD-fed FVB/N mice had fewer crown-like structures - histologic arrangements composed of macrophages and dead or dying adipocytes that define white adipose tissue inflammation (Fig. 2f) [32]. Likewise, the peritumoral adipose tissue of obese wild-type C57BL/6 J mice had higher expression of monocyte chemoattractants such as *Ccl2* (Fig. 2g), which is in agreement with human data [5]. Overall, these results indicate that in our experimental model, obesity promotes primary tumor growth and metastasis formation, while HFD in the absence of obesity is not sufficient to do this.

### Obesity reduces angiogenesis and promotes hypoxia in the primary site

**Fig. 3:**
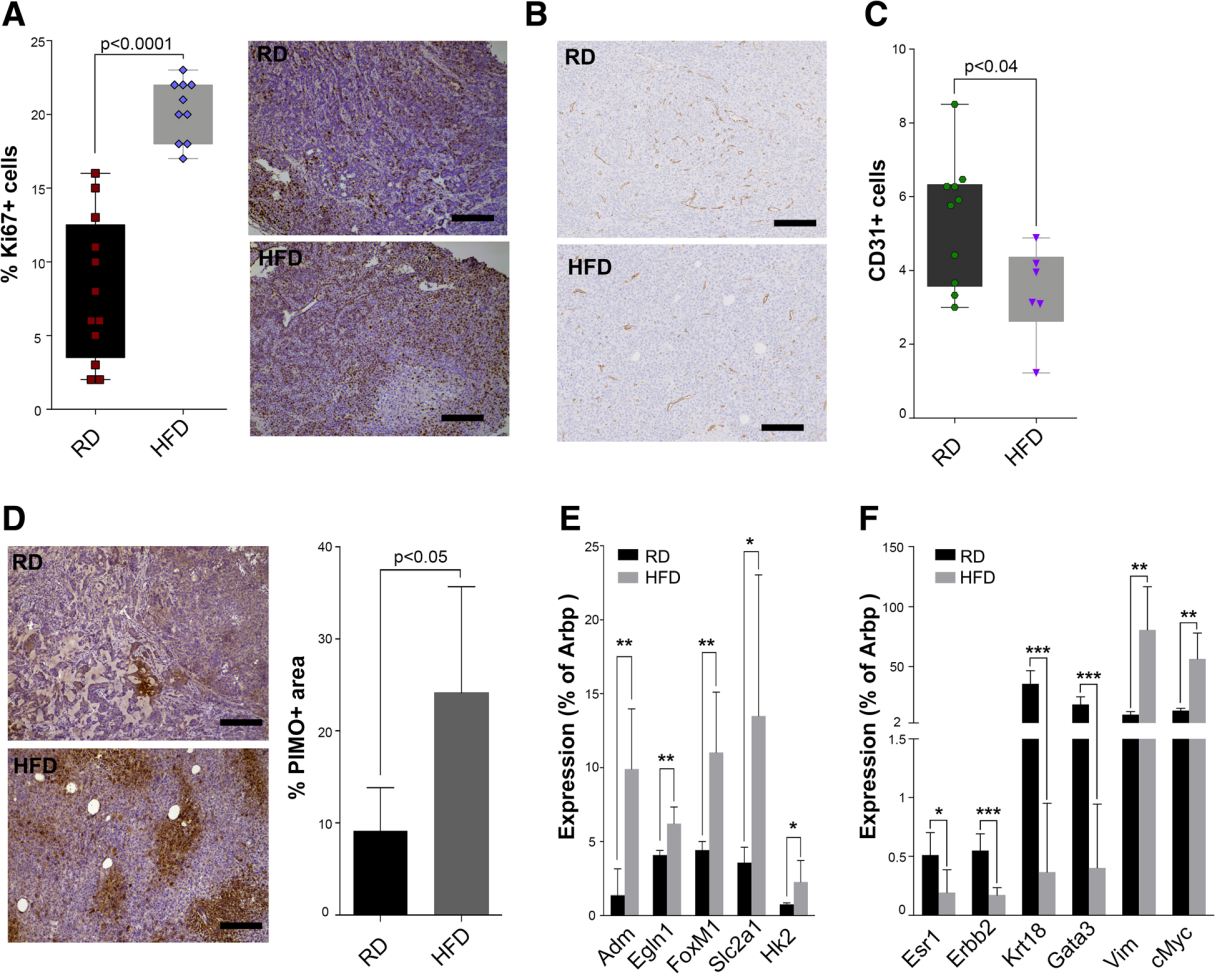
Increased tumor hypoxia in obese mice. Tumors in mice fed a high-fat diet (HFD) have higher Ki67+ counts (**a**, scalebar 200 um). CD31 immunohistochemical analysis shows lower vessel density in tumors from HFD mice (**b**, scalebar 200 uM). This is supported by fluorescence-activated cell sorting quantification (**c**). Pimonidazole (PIMO) staining in mouse fed a regular diet (RD) or a HFD demonstrate greater hypoxic areas in tumors from HFD mice (**d**, scalebar 200 uM). Quantitative PCR analyses on RD and HFD tumors show upregulation of hypoxia inducible factor 1 alpha (HIF1α) targets (**e**, *n* = 5) and faster tumor progression (**f**, *n* = 5). Arbp, attachment region binding protein

We then aimed at investigating possible reasons for faster primary tumor progression in obese mice. Not surprisingly, we observed an increase in the fraction of proliferating cancer cells in early-stage tumors in obese mice (Fig. 3a). To study the association between obesity and faster tumor progression, we next analyzed tumor angiogenesis. A number of reports show that in obesity, angiogenesis cannot cope with adipose tissue growth [33–37]. We hypothesized that this might be mirrored in tumors, since the mammary gland is mainly composed of adipose tissue and tumors are surrounded by and in close contact with adipose tissue. In agreement with this, we found fewer vessels and lower fractions of CD31^+^ cells in tumors in obese mice (Fig. 3b, c and Additional file 2: Figure S2A). To understand the impact of decreased angiogenesis on oxygen levels in tumors in HFD-fed mice, we injected mice with pimonidazole and found higher hypoxic regions in tumors in obese mice (Fig. 3d). Furthermore, hypoxia in tumors from obese mice led to the accumulation of HIF1α (Additional file 3: Figure S2B), which consequently activated the transcription of specific hypoxia-target genes (Fig. 3e). Interestingly, HIF1α is known to be highly activated in triple-negative breast cancer (TNBC) [38, 39], a subset of aggressive breast cancers most of which are high-grade and present a high risk of metastasis and recurrence [40]. Indeed, histological analyses revealed that in obese mice the tumor mass was less differentiated, more often lacking glandular structures and possessing bigger nuclei (Additional file 3: Figure S2C). In addition, Py230 tumors in obese mice showed a consistent reduction in ERα, human epidermal growth factor receptor 2 (HER2), GATA3 and cytokeratin 18 and a gain in vimentin and c-Myc expression (Fig. 3f and Additional file 3: Figure S2D), suggestive of differentiation into more aggressive TNBC tumors. Overall, our results indicate that obesity causes reduced angiogenesis and triggers hypoxia in primary tumors, which promotes tumor progression.

### The obese primary tumor microenvironment stimulates the expansion of metastasis-initiating cells

**Fig. 4:**
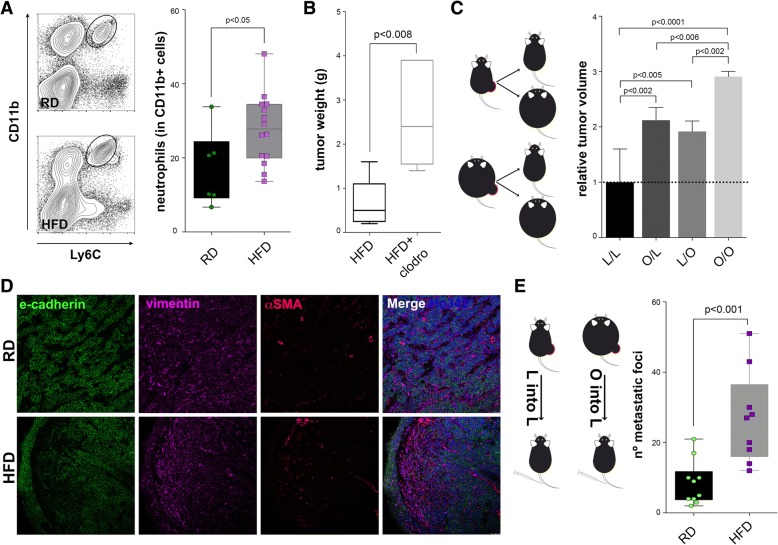
Microenvironmental effects on tumor cells. Tumors from mice fed a high-fat diet (HFD) contain greater numbers of neutrophils (**a**, *n* = 6 regular diet (RD), *n* = 14 HFD). Clodronate liposomes treatment increases tumor weight (**b**, *n* = 5 RD, *n* = 4 HFD). Cross-transplantation experiments reveal that the effects of the obese microenvironment on tumor cells are permanent (**c**, *n* = 14). Immunofluorescent staining of tumors show increased epithelial-mesenchymal transition (EMT) features in HFD groups (**d**). Tumor cells in tumors from mice fed RD or HFD injected intravenously into RD mice show different metastasis initiating potential (**e**, *n* = 19). Clodro, clodronate liposomes; αSMA, alpha smooth muscle actin; l, lean; O, obese

Given the essential involvement of inflammation in obesity [41], we next aimed at understanding how decreased angiogenesis and hypoxia modulate the immune compartment in the primary tumor. FACS analyses revealed that tumors from HFD mice contained 23% less CD11b^+^F4/80^+^ macrophages (Additional file 4: Figure S3A), which are mostly M1 macrophages in the C57BL/6 model (Additional file 4: Figure S3B). In contrast, the population of CD11b^+^F4/80^−^ cells showed a 31% increase in tumors from HFD mice. This population consists of CD11b^+^Ly6C^med^Gr1^+^ neutrophils and CD11b^+^Ly6C^high^ monocytes (Fig. 4a). We confirmed these results by performing western blot analyses and found increased CD11b protein in tumor tissue lysates from HFD mice, compared to tumors from RD mice (Additional file 4: Figure S3C). Of note, this increase was not seen in tumors grown in C57BL/6 Rag2−/− or FVB/N mice fed with HFD diet (Additional file 4: Figure S3D and E), which underscores again the immunological differences between these strains. We then reasoned that if fast-growing tumors in HFD-fed mice contain fewer M1 macrophages and more tumor associated neutrophils (TANs) compared to tumors growing in RD-fed mice, macrophage may be protective against tumor growth. To test this hypothesis, we treated mice with clodronate liposomes to deplete macrophages. Indeed, clodronate liposome treatment boosted primary tumor growth in HFD-fed mice (Fig. 4b) and did not reduce metastasis (Additional file 4: Figure S3F). These results suggest that in our model, macrophages do not contribute to promote tumor progression and metastatic spread, regardless of their essential involvement in obesity.

In order to evaluate the importance of the effects of the microenvironment on the tumor cells, we performed cross-transplantation of tumor cells from HFD-fed and RD-fed into RD-fed and HFD-fed mice, respectively. Interestingly, we observed that tumor cells derived from obese mice grew faster in lean recipient mice compared to cells derived from lean mice (Fig. 4c). As expected, grafting into obese mice further boosted growth of both transplanted cell populations. These results uncoupled immediate microenvironmental effects from tumor cell effects and indicate that the obese tumor microenvironment exerts contextual and sustained effects on tumor cells.

Neutrophils are known to migrate to ischemic tissues and to contribute to the epithelial-to-mesenchymal transition (EMT) [42]. EMT is a process involved in invasion and metastasis and produces cancer stem cells (CSC) [43], a subpopulation of cells that we and others have previously shown to lead metastatic colonization [15]. Indeed, tumors from HFD mice consistently lost E-cadherin and had an increase in N-cadherin and vimentin, three hallmarks of EMT (Fig. 4d and Additional file 5: Figure S4A). This effect was not observed in FVB/N tumors (Additional file 5: Figure S4B). In agreement with TANs being associated with EMT, we identified strong correlation between the expression of CD11b and N-cadherin and anti-correlation with E-cadherin in primary tumors (Additional file 5: Figure S4D).

All these assays were performed with equal-sized tumors to avoid potential confounding effects due to more rapid tumor growth in HFD-fed mice (Additional file 5: Figure S4C). EMT is a distinctive feature of claudin-low tumors, a particular subset of TNBC that is enriched in CSC-related genes [44]. Since aggressive subtypes of breast cancer such as TNBC and basal-like tumors are associated with mutations in p53 [23, 45–47], we next stained for p53, a surrogate marker for its mutational status. Our results indicate that tumors in obese mice have a higher number of p53-positive cells (Additional file 5: Figure S4E). As a consequence, they also show significantly lower levels of p21(WAF1/CIP1), an important target of p53 responsible for cell cycle arrest (Additional file 5: Figure S4A).

Claudin-low tumors are also characterized by a loss of cell-cell junction proteins. Therefore, we next performed qPCR analyses in tumors from RD and HFD mice using a number of genes from the cell-cell junction organization gene set M820 from the MSigDB database [48], as previously described [23]. The results confirmed that the obese microenvironment triggers a process that leads to the rapid expansion of claudin-low tumors (Additional file 5: Figure S4F).

Finally, to test whether the effects of obesity on the primary tumor are essential for the late steps of cancer metastasis, we digested tumors from RD and HFD mice and injected 5 × 10^5^ tumor cells via the tail vein into tumor-free, RD mice. Our results demonstrate that tumor cells derived from obese mice metastasize more to the lungs compared to cells derived from lean mice (Fig. 4e), i.e. tumors from obese mice contain more CSC with metastasis-initiating capacity. Our data provide direct evidence that the primary tumor microenvironment of obese mice generates more tumor cells with lung metastasis-initiating capacity.

## Discussion

To date, the link between obesity and worse outcomes observed in patients with breast cancer remains poorly understood, mainly due to the lack of experimental studies based on mouse models of metastasis that explore the full metastatic cascade. In this study, we used orthotopic, syngeneic models of spontaneous breast cancer metastasis and have discovered a novel experimental link between obesity and tumor progression to metastasis; collectively, our results show that the interactions between hypoxia, elements of the tumor microenvironment (likely neutrophils) and tumor cells ultimately orchestrate a shift towards TNBC/claudin-low tumors and a consequent increase in metastasis-initiating cells within primary tumors in obese mice. Overall, our data provide an experimental link with clinical observations describing higher TNBC rates in obese patients [2, 49]. Moreover, premenopausal and postmenopausal, overweight and obese patients with breast cancer are generally at higher risk of recurrence and resistance to therapy [1, 2, 12, 50–52]. Biganzoli and collaborators used data from the prospective “three-arms” trial with very long follow up to show that the patient’s BMI at diagnosis is associated with specific recurrence patterns over time [53]. They observed for example that obese patients present with at least two peaks of recurrences, one early and one late. Our results showing TNBC features and increased CSC content in tumors in obese mice might explain the higher risk of recurrence and resistance to therapy observed in overweight and obese humans, although this hypothesis requires further studies to be confirmed.

Interestingly, recent work suggests that higher neutrophil infiltration in the lungs of obese mice results in higher metastatic burden [13]. While in our setting primary tumor hypoxia could also be responsible for the generation of a neutrophilic premetastatic niche in the lungs [54], we here showed that the events in the primary tumor promote increased metastasis in obese mice without the need for preconditioning the distant metastatic site. Accordingly, in secondary transplants obese primary tumors have higher metastatic potential regardless of the host in which they are grafted. The different conclusions between this work [13] and ours are likely to be explained by experimental differences, the most significant of which is the use of orthotopic models of spontaneous lung metastasis, which is the only strategy that allows study of the whole metastatic cascade. In addition, our obese mice were ovariectomized, thereby better mimicking postmenopausal obesity in patients. In short, while our results do not exclude additional metastasis-promoting effects in the secondary site, they clearly reveal important effects of postmenopausal obesity on the primary tumor which are critical for metastatic spread and colonization. We therefore think that our model provides a more clinically relevant approach to unravel the effects of obesity on breast cancer progression.

Due to the lack of ovarian-derived estrogens, postmenopausal women are more prone to increases in their BMI. We show that in our model this is not due to dietary factors but, similarly to humans, it is linked to each individual’s susceptibility to become obese [26]. Indeed, to address the importance of the diet in the progression of breast cancer, we used obesity-resistant M2/Th2 FVB/N mice and demonstrated that the diet alone, i.e. in the absence of obesity, is not sufficient to affect tumor growth.

Local estrogen production has also been linked to the increased risk of breast cancer and contributes to progression in postmenopausal women, given that after menopause the production of estrogens is thought to occur mainly in the adipose tissue [55]. However, we were not able to detect aromatase transcripts in the adipose tissue or the tumors of RD or HFD mice (data not shown), which rules out potential effects of local estrogen production on tumor growth in obese animals.

Obesity is characterized by low-grade chronic inflammation. Our results indicate correlation between the infiltration of neutrophils in the primary tumor and the acquisition of a more mesenchymal phenotype by tumor cells. In contrast, Kolb and collaborators found that the inflammasome of macrophages in primary tumors in obese mice is responsible for triggering angiogenesis through expression of vascular endothelial growth factor A (VEGFA), consequently boosting primary tumor growth [10]. Our results differ in that we did not see increased macrophage content in tumors from obese mice, and we observed a reduction in vessel density with a concomitant increase in hypoxia. We argue that this decrease in vascularization is the same as observed in the adipose tissue during obesity [56], and we suggest that higher proliferative rates may be the result of p53 dysregulation. Notwithstanding the differences between Kolb et al. and our group, it is worth noting that in our experimental setting elimination of macrophages by treatment with clodronate liposomes did not reduce tumor growth but rather the opposite. In addition, we and others have observed that clodronate treatment reduces body weight in obese mice, which is consistent with an obesity-promoting effect of M1 macrophages [57]. Finally, it is known that during obesity there is an increase in neutrophil recruitment in the adipose tissue, which mediates insulin resistance [58, 59]. Overall, our results indicate that obesity-associated macrophages play a crucial role in stimulating the growth of the adipose tissue, but they have antagonistic effects on cancer progression. We here suggest that other immune cells, such as neutrophils, might be involved in primary tumor progression in obesity. Our model might prove useful in identifying further key factors relevant to breast cancer progression in obesity and to evaluate potential therapeutic strategies.

## Conclusions

In summary, we have found that decreased vascularization in the primary tumors of postmenopausal obese mice triggers hypoxia, neutrophil infiltration and EMT, leading to the expansion of TNBC/claudin-low tumors and an increase in metastasis-initiating cells. These results provide an explanation for the higher incidence of metastasis and higher ratio of TNBC observed in obese patients with breast cancer and challenge the recent notion that tumor-cell extrinsic factors in the secondary site are clinically relevant to these patients.

## Additional files

Additional file 1: **Table S1**. (DOCX 45.3 kb)

Additional file 2: **Figure S1.** Scheme of the experimental procedure (A). Body weight comparison between ovariectomized (*n* = 12 RD, *n* = 12 HFD) and non-ovariectomized (*n* = 5 RD, *n* = 5 HFD) mice (B). Body weight of ovariectomized RD and HFD mouse groups after 13 weeks of diet (C; *n* = 29 RD and *n* = 35 HFD). Data distribution of mouse weight in RD (D) and HFD (E) groups. Number of metastases seeded by same-sized tumors in RD and HFD mice (F and G). Error bars in panel B indicate SEM. (TIF 2261 kb)

Additional file 3: **Figure S2.** CD31 staining in C57BL/6 Rag2−/− mice show no differences between tumors from RD and HFD mice when they are grown in C57BL/6 Rag2−/− hosts (A, scalebar = 100uM). HIF1a staining in wild-type (wt) and C57BL/6 Rag2−/− mice show that hypoxia is increased in tumors from wt obese mice, while it is not changed in tumors in HFD-fed C57BL/6 Rag2−/− mice (B, scalebar 100 uM). HE staining of RD and HFD tumors showing enlarged nuclei and less packed chromatin in the latter (C, scalebar 50 um). IHC analyses in tumor samples show faster progression in HFD compared to RD tumors (D, *n* = 5, scalebar 50 um). (JPG 3325 kb)

Additional file 4: **Figure S3.** FACS analyses show lower percentages of F4/80+ macrophages in the CD11b + compartment in Py230 HFD tumors (A, *n* = 18 RD, *n* = 20 HFD). Percentages of M1 (F4/80 + CD206-) and M2 macrophages (F4/80 + CD206+) identified in the CD11b + compartment of C57BL/6 and FVB/N mice (B, n = 3 RD, *n* = 7 HFD for C57BL/6 and *n* = 4 RD, *n* = 4 HFD for FVB/N). Normalized western blot analysis for CD11b in Py230-C57BL6 tumors (C; *N* = 8), Py230-C57BL/6;Rag2−/− tumors (D; *N* = 9) and PyMT-FVB/N tumors (E; *n* = 8). Clodronate liposomes treatment increases metastasis (F, *n* = 5 RD, *n* = 6 HFD). (TIF 1676 kb)

Additional file 5: **Figure S4.** Western blot analysis for E-cadherin, N-cadherin, p21, and HIF1a in Py230-C57BL/6 tumor lysates of RD and HFD (A). Western blot analysis for E-cadherin in PyMT tumors grown in FVB/N mice (B). Tumor weight of groups used for qPCR and western blot analyses (C, *N* = 10). CD11b strongly correlates with N-cadherin and anti-correlates with E-cadherin (D, *N* = 10). IHC on RD vs HFD tumors show nuclear p53 accumulation in the latter (E, scalebar 100 uM). qPCR analyses on Py230 RD and HFD tumors show significant downregulation of claudins and other cell-cell junction genes (F, *n* = 5). (TIF 9127 kb)

## Abbreviations

ANOVA: Analysis of variance
BMI: Body mass index
CSC: Cancer stem cell
EMT: Epithelial to mesenchymal transition
FACS: Fluorescence-activated cell sorting
HER2: Human epidermal growth receptor 2
HFD: High-fat diet
HIF1α: Hypoxia inducible factor 1 alpha
IHC: Immunohistochemistry
PBS: Phosphate-buffered saline
RD: Regular diet
TAN: Tumor associated neutrophil
TNBC: Triple-negative breast cancer
