# Supplementary material for: Obesity promotes the expansion of metastasis-initiating cells in breast cancer
Source: Breast Cancer Res. 2018 Sep 4;20:104. doi: 10.1186/s13058-018-1029-4 (PMC6123990; doi:10.1186/s13058-018-1029-4)
Supplement: Supplementary file 1 — Table S1. (DOCX 45.3 kb) [file 13058_2018_1029_MOESM1_ESM.docx]

**Additional file 1: Table S1:**

| **ID** | **Forward 5’-3’** | **Reverse 5’-3’** |
| --- | --- | --- |
| Esr1 | TCCGGCACATGAGTAACAAA | CCAGGAGCAGGTCATAGAGG |
| Erbb2 | GAAGCCAGAGCATCTCCAAG | TCTGGCCATGCTGAAATGTA |
| Krt18 | CGAGGCACTCAAGGAAGAAC | AATCTGGGCTTCCAGACCTT |
| Gata3 | GCTACGGTGCAGAGGTATCC | AGAGATCCGTGCAGCAGAG |
| Slc1a2 | TTCTCTGTCGGCCTCTTTGT | GAGAAGCCCATAAGCACAGC |
| Hk2 | GGCAGAGATGTGGTGGATCT | TCATTCACCACAGCCACAAT |
| c-Myc | CCTAGTGCTGCATGAGGAGAC | GATGGAGATGAGCCCGACT |
| Ccl2 | CCCAATGAGTAGGCTGGAGA | TCTGGACCCATTCCTTCTTG |
| Il6 | ATGGATGCTACCAAACTGGAT | TGAAGGACTCTGGCTTTGTCT |
| Arbp | GATTCGGGATATGCTGTTGG | GTTCTGAGCTGGCACAGTGA |
| Cldn1 | GATGTGGATGGCTGTCATTG | CCTGGCCAAATTCATACCTG |
| Cldn7 | AGCATGTTCCTGGATTGGTC | CCAGAAGGACCAGAGCAGAC |
| Cldn10 | AAAGTCGGAGGCTCAGATCA | ACAGCCTGTCATGGAACACA |
| Cldn14 | AAGACTGTTGAAGCCGCAGT | CGCAGGAAGCCTCATACATC |
| F11r | TATGATCCTGGGCTCTTTGG | GGGAGAGGAGAAGCCAGAGT |
| Lama3 | GGGTGTGACCAAAAAGTGCT | GAGGGCACATCCAAGTTTGT |
| Arhgef6 | AGGCTGCCTACTGAACCTCA | TGCACCTTGATTTTCCATGA |
| Col17a1 | CTGGATTAGGCAAGGCTGAG | CTTGACTCCCCATGTCACCT |
| Lamc2 | AGTTCTGCCCGAAGATCAGA | CACGCGGTAGTCAAAAGACA |
| Pard6g | TGACGACAACTTCTGCAAGG | AGCACCTTTTTCTTCCGTGA |
| Adm | CAGAGCATCGCCACAGAATG | ACGACTTAGCGCCCACTTAT |
| Egln1 | TTGCTGACATTGAACCCAAA | CTCGCTCATCTGCATCAAAA |
| FoxM1 | CCAAGGCAAAGACAGGAGAG | CCAAGCCACTGGATATTGGT |
