# Supplementary figures and images for: Obesity promotes the expansion of metastasis-initiating cells in breast cancer
Source: Breast Cancer Res. 2018 Sep 4;20:104. doi: 10.1186/s13058-018-1029-4 (PMC6123990; doi:10.1186/s13058-018-1029-4)

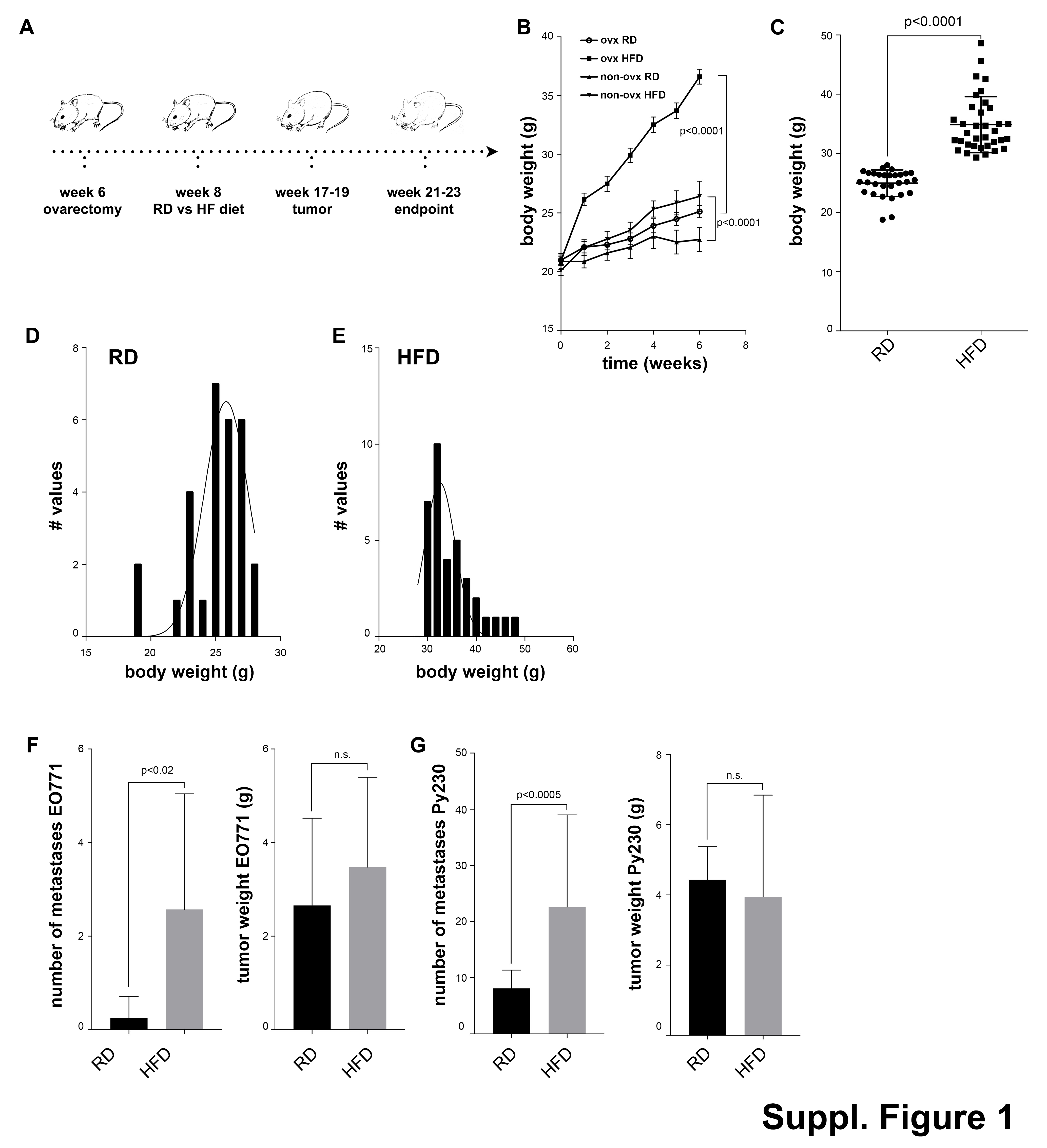

Supplement: Supplementary file 2 — Figure S1. Scheme of the experimental procedure (A). Body weight comparison between ovariectomized (n = 12 RD, n = 12 HFD) and non-ovariectomized (n = 5 RD, n = 5 HFD) mice (B). Body weight of ovariectomized RD and HFD mouse groups after 13 weeks of diet (C; n = 29 RD and n = 35 HFD). Data distribution of mouse weight in RD (D) and HFD (E) groups. Number of metastases seeded by same-sized tumors in RD and HFD mice (F and G). Error bars in panel B indicate SEM. (TIF 2261 kb) [file 13058_2018_1029_MOESM2_ESM.tif]

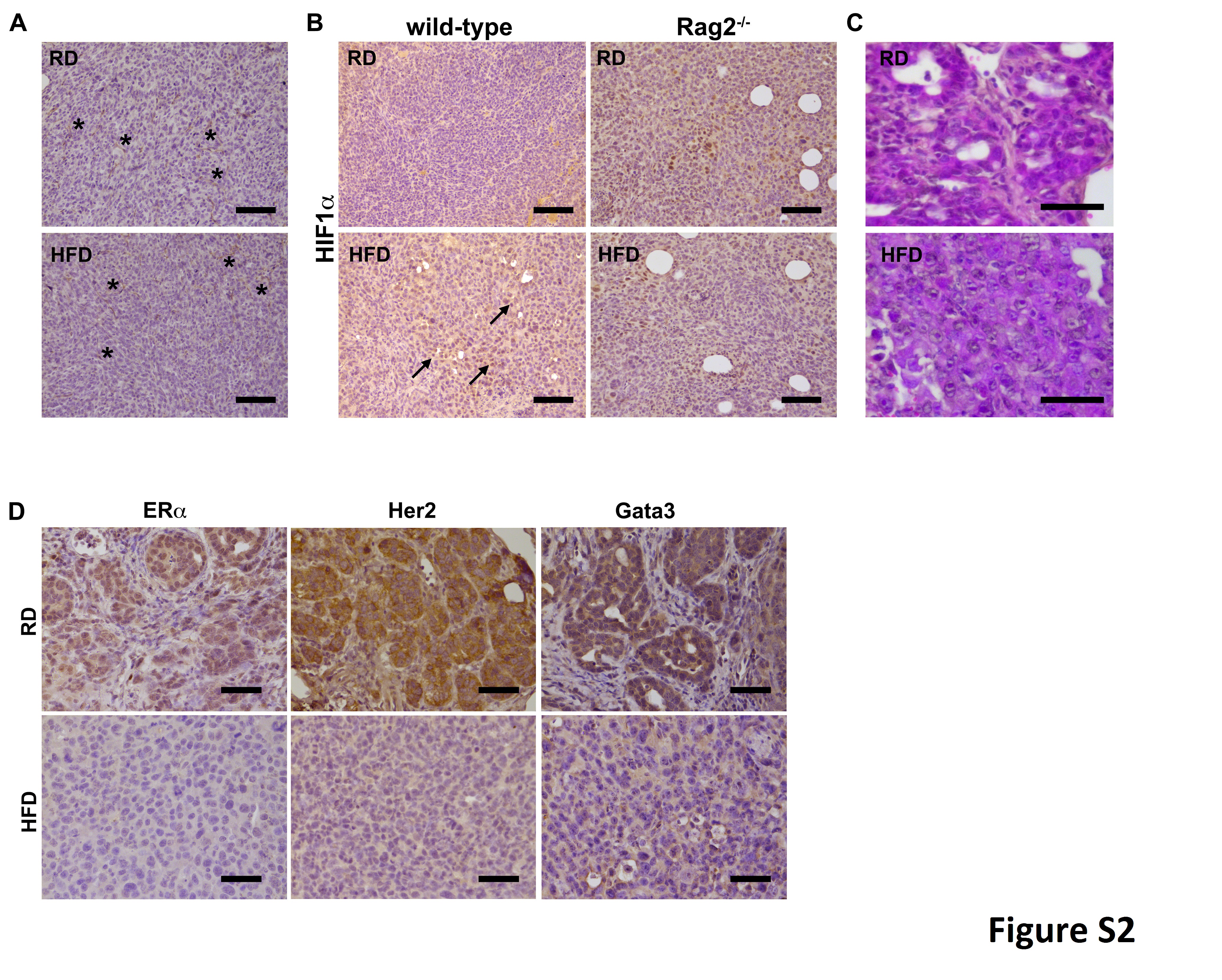

Supplement: Supplementary file 3 — Figure S2. CD31 staining in C57BL/6 Rag2−/− mice show no differences between tumors from RD and HFD mice when they are grown in C57BL/6 Rag2−/− hosts (A, scalebar = 100uM). HIF1a staining in wild-type (wt) and C57BL/6 Rag2−/− mice show that hypoxia is increased in tumors from wt obese mice, while it is not changed in tumors in HFD-fed C57BL/6 Rag2−/− mice (B, scalebar 100 uM). HE staining of RD and HFD tumors showing enlarged nuclei and less packed chromatin in the latter (C, scalebar 50 um). IHC analyses in tumor samples show faster progression in HFD compared to RD tumors (D, n = 5, scalebar 50 um). (JPG 3325 kb) [file 13058_2018_1029_MOESM3_ESM.jpg]

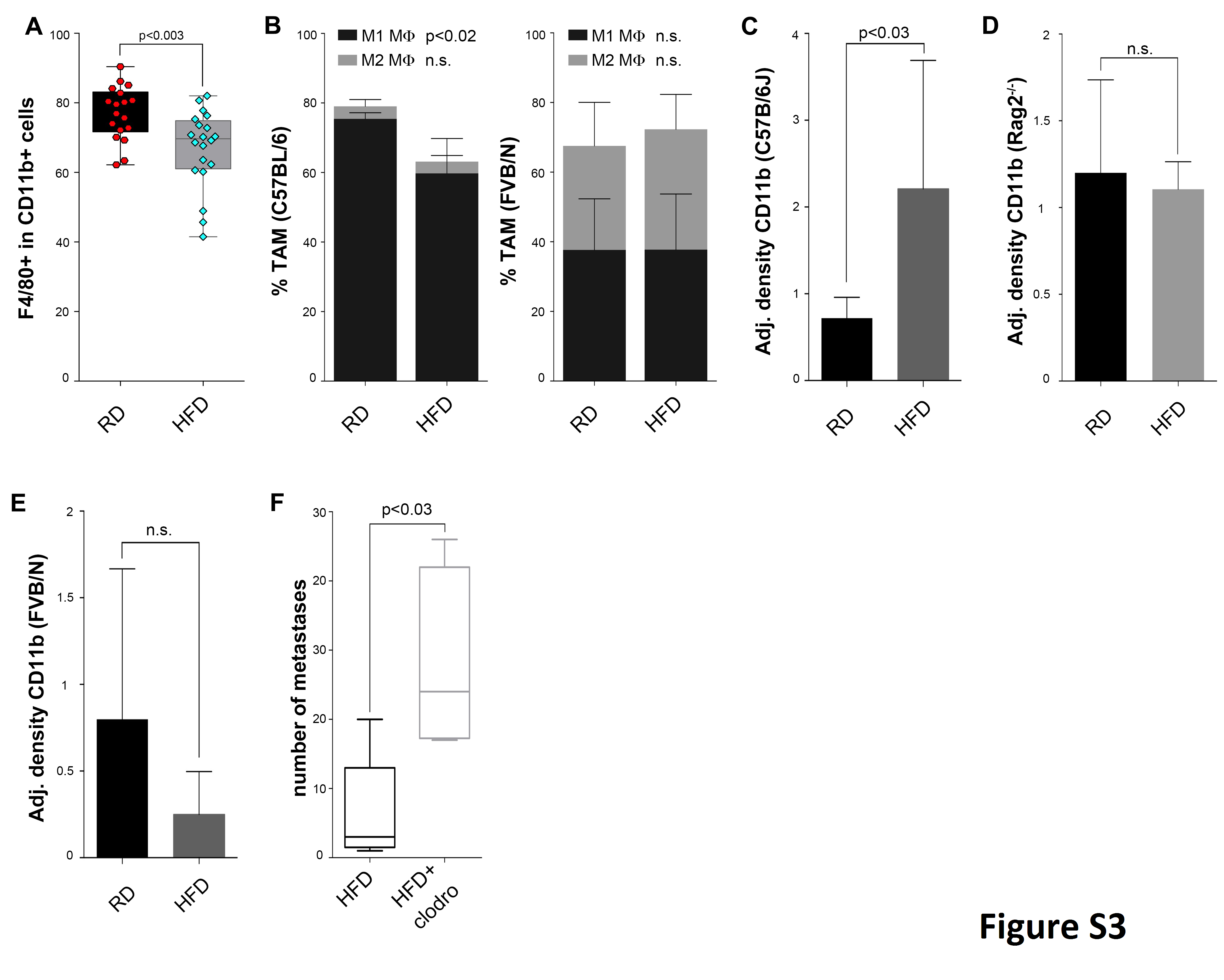

Supplement: Supplementary file 4 — Figure S3. FACS analyses show lower percentages of F4/80+ macrophages in the CD11b + compartment in Py230 HFD tumors (A, n = 18 RD, n = 20 HFD). Percentages of M1 (F4/80 + CD206-) and M2 macrophages (F4/80 + CD206+) identified in the CD11b + compartment of C57BL/6 and FVB/N mice (B, n = 3 RD, n = 7 HFD for C57BL/6 and n = 4 RD, n = 4 HFD for FVB/N). Normalized western blot analysis for CD11b in Py230-C57BL6 tumors (C; N = 8), Py230-C57BL/6;Rag2−/− tumors (D; N = 9) and PyMT-FVB/N tumors (E; n = 8). Clodronate liposomes treatment increases metastasis (F, n = 5 RD, n = 6 HFD). (TIF 1676 kb) [file 13058_2018_1029_MOESM4_ESM.tif]

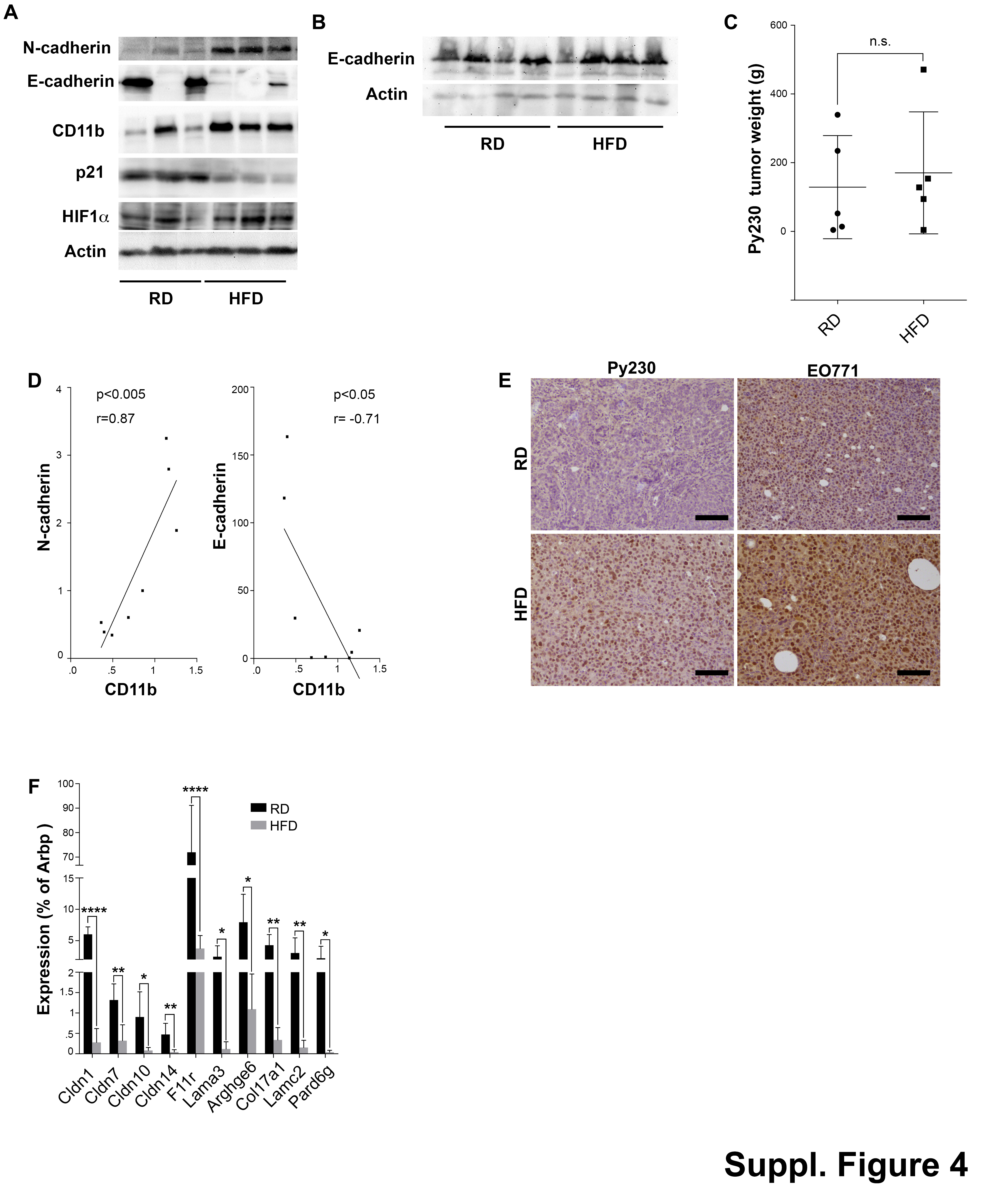

Supplement: Supplementary file 5 — Figure S4. Western blot analysis for E-cadherin, N-cadherin, p21, and HIF1a in Py230-C57BL/6 tumor lysates of RD and HFD (A). Western blot analysis for E-cadherin in PyMT tumors grown in FVB/N mice (B). Tumor weight of groups used for qPCR and western blot analyses (C, N = 10). CD11b strongly correlates with N-cadherin and anti-correlates with E-cadherin (D, N = 10). IHC on RD vs HFD tumors show nuclear p53 accumulation in the latter (E, scalebar 100 uM). qPCR analyses on Py230 RD and HFD tumors show significant downregulation of claudins and other cell-cell junction genes (F, n = 5). (TIF 9127 kb) [file 13058_2018_1029_MOESM5_ESM.tif]
